# IUPESM: the international umbrella organisation for biomedical engineering and medical physics

**DOI:** 10.2349/biij.3.3.e56

**Published:** 2007-07-01

**Authors:** JH Nagel

**Affiliations:** Department of Biomedical Engineering, University of Stuttgart, Stuttgart, Germany

**Keywords:** IUPESM, IFMBE, IOMP, ICSU, biomedical engineering, medical physics

## Abstract

An account of the development, aims and activities of the International Union for Physical and Engineering Sciences in Medicine (IUPESM) is presented. Associations with the International Council of Science (ICSU) and the World Health Organization (WHO) are leading to exciting new projects towards improving global health, healthcare, quality of life and support of health technologies in developing countries.

## HISTORY

The 20th century was an inspirational period for physical and engineering sciences applied to medicine. The seeds for the rapid growth of medical physics and biomedical engineering in healthcare were already sown in the closing decade of the 19th century by three important discoveries: x-rays by Wilhelm Röntgen in Germany in 1895, radioactivity by Henri Becquerel in France in 1896, and the electron by JJ Thompson in England in 1897. As with other aspects of technological development, the underlying principles elucidated by physicists were soon turned into practical applications through the skill of engineers. As we enter the 21st century, medical physicists and biomedical engineers continue to play an essential role in delivering modern, effective healthcare in a wide variety of ways. The work of these dedicated health professionals takes place in hospitals, research laboratories, industrial companies, academic institutions, and governmental organisations.

In the early second half of the last century, two international scientific and engineering organisations with a large overlap of interests [1] were founded, both with the intent to support the newly forming national societies in the fields of biomedical engineering and medical physics in their missions to strengthen research and practical applications of their disciplines to improve the health, healthcare and quality of life of all human beings. These two international organizations were the International Federation for Medical and Biological Engineering (IFMBE) founded in 1959, and the International Organization for Medical Physics (IOMP) founded in 1963. In the following years, many other international organisations appeared with objectives similar to those of the IFMBE and the IOMP, spurred by the rapid growth in the scope and increasingly important interaction between engineering as well as science and medicine, coupled with the perception that the IFMBE and the IOMP could not cover the whole field. Many sub-disciplines of medical and biological science, engineering as well as medical physics formed their own organisations, including those for biomechanics, biomaterials, artificial organs, computing in medicine, medical informatics, hospital engineering, nuclear medicine, radiation research, radiation protection and magnetic resonance in medicine. Some developed cooperation with the IFMBE and IOMP, while others appeared to be competing.

The Federation (www.ifmbe.org), however, considered itself unique in its general coverage of biomedical science and engineering, and firmly believed that this breadth should be retained and that it should work towards building links with and between specialist bodies to encourage interaction and integration [2]. The Federation also considered it important to build a strong bond with physicists working in the medical sector and viewed the IOMP (www.iomp.org) as a potential sister organisation.

There was considerable overlap between the activities of the two organisations, and the discussion of a possible collaboration started in the 1970s. Opinions about cooperation differed among the member countries. This was at least partially due to the fact that in some countries the membership in societies for engineers and those for physicists almost totally overlapped, or there was only one common society for engineers and physicists, while in other countries the two groups formed entirely separate associations.

After several years of preliminary explorations, the IFMBE collaborated with the IOMP in its Third International Conference of Medical Physics, held in 1973 in Göteborg, Sweden. In that year representatives from the two organisations met and discussed the possibility of collaborating or merging.

In order to create the opportunity to exchange views between biomedical engineers and medical physicists, to learn from each other and to think about how to improve cooperation between IFMBE and IOMP, the two organisations held their major international conferences together for the first time in 1976. The two meetings were still separated, but held in immediate succession in Ottawa, Canada, and the leaders of the associations met officially to exchange views on a closer relationship. Agreement was reached in principle that an umbrella organisation should be formed and that the IFMBE and the IOMP should hold joint conferences, although each association would retain its autonomy.

In 1979, the conferences were completely merged and held in Jerusalem as the “Combined Meeting of the XII. International Conference on Medical and Biological Engineering and V. International Conference on Medical Physics”, a very special event that triggered the foundation of the long-discussed umbrella organisation for the IOMP and IFMBE: the International Union for Physical and Engineering Sciences in Medicine (IUPESM) in 1980. The joint conference became the “World Congress for Medical Physics and Biomedical Engineering” and was first held under this name in 1982 in Hamburg, Germany. Since then, the World Congress has been organised every three years, the next one being scheduled for 2009 in Munich, Germany (www.wc2009.org).

The IUPESM now represents more than 140,000 professionals in over 100 countries. In spite of the difference in membership numbers between IFMBE (>120,000) and IOMP (>20,000), the Union operates based on the principle that there should be equitable sharing between the two organisations. Thus, the surplus generated by the World Congresses does not contribute to the IUPESM income at all, but is shared between the organising national societies, the IFMBE and the IOMP.

The IUPESM objectives, as stated in its constitution, are to contribute to physical and engineering science in medicine, to organise international cooperation, to coordinate activities by holding conferences, and to represent the professional interests and views of engineers and physical scientists in the healthcare community.

As for all other associations, the key question to be answered when justifying their existence is what the benefits are for their members and how much they contribute to a positive development of society as a whole, with IUPESM focusing on the health and well-being of the people. For many years, the only major activity of the IUPESM was the World Congress. New fields of cooperation between IFMBE and IOMP, such as the launch of the book *Series in Medical Physics and Biomedical Engineering* in 1994, remained largely outside IUPESM. As a logical consequence, members of both constituent bodies started asking whether their umbrella organisation was actually worth being sustained. Such thoughts were nourished by occasional problems that arose due to differences in policy and emphasis, though agreement has always been reached based on the belief that collaboration is more important than controversy. By the end of the 1990s, however, there was only one issue that prevented the Union from breaking apart: the common desire of the IOMP and the IFMBE to be recognised by the International Council of Science (ICSU) as scientific organisations. For many years, the necessary cooperation between IFMBE and IOMP to reach this goal was the bond that kept IUPESM together. Fortunately, IUPESM was admitted to ICSU as a full member in 1999 after many years of hard work by the IUPESM officers, in particular Keith Boddy (IUPESM President 1997-2000), and persistent, convincing discussions between the constituent societies of IFMBE and IOMP with the national members of ICSU, which were mostly the national academies of science. Since its admission to ICSU, the IUPESM has consistently engaged in the establishment of ICSU-sponsored research projects related to health.

Though the IFMBE has a long history of working with the World Health Organization (WHO), mainly in support of developing countries, it only started in this millennium to develop really strong activities on professional issues and to become a powerful international player in the area of public health, the global improvement of the health workforce and in advocacy for improved patient safety. Recently the IOMP, too, adopted a more proactive approach to support developing countries, accepting a responsibility towards those nations that may deviate somewhat from past priorities. These developments made it possible for the IUPESM, at its last Council meeting in 2006, to decide on establishing an official relationship and close cooperation with WHO. As a first initiative in this new partnership, IUPESM decided to launch a *Health Technology and Training Task Group (HTTTG)* with the goal of helping developing countries to improve their healthcare systems by introducing appropriate health technologies and training for their health workforce.

At the IUPESM meeting in Seoul during the World Congress 2006, the General Assembly also decided to accept new members to the Union, to further strengthen collaboration between IFMBE and IOMP committees and to start new initiatives in cooperation between the two organisations. So it appears that after 25 years, the liaison between IFMBE and IOMP has begun to bear fruits beyond the common Congress. The IUPESM is starting a new life as an active international representative of the biomedical/clinical engineering and medical physics community.

## OBJECTIVES

The principal objectives of the IUPESM are to contribute to the advancement of physical and engineering science in medicine for the benefit and wellbeing of humanity; to organise international cooperation and promote communication among those engaged in healthcare science and technology; to coordinate activities of mutual interest to engineering and physical science within the healthcare field, including international and regional scientific conferences, seminars, working groups, regional support programs and scientific and technical publications; and to represent the professional interests and views of engineers and physical scientists in the healthcare community.

In order to further attain these objectives, the IUPESM shall, according to its statutes, be empowered to:

1. collaborate with other international scientific and professional bodies;
2. establish committees, commissions, working groups and other bodies for purposes within its mandate;
3. organise and coordinate international meetings or conferences for the constituent associations within the IUPESM, including the triennial World Congress on Medical Physics and Biomedical Engineering;
4. represent the IUPESM members in the International Council of Scientific Unions, in accordance with the statutes of ICSU;
5. disseminate, promote and/or develop standards of practice in the fields of medical physics and biomedical engineering in order to enhance the quality of health care worldwide;
6. assist developing countries to achieve appropriate levels of science and technology in medical physics and biomedical engineering;
7. provide suitable channels for the exchange of information between nations.

Points (5) and (6) are rather unusual for a scientific union and stress the high responsibility that the IUPESM has assumed for professional issues, the enhancement of healthcare worldwide and their dedication in assisting the developing countries to achieve high quality healthcare for their people. Led by Barry Allen and Joachim Nagel, IUPESM has stepped up its efforts towards attaining these objectives with the recent launch of its Health Technology and Training Task Group.

The Union has also established key programs, which are complementary to, and symbiotic with, those of ICSU. They include Public and Governmental Understanding of Health Sciences; Education, Training and Continued Professional Development for the 21st Century and Global Biomedical Information Networking for developing countries for which a Global On-line Medical Physics Textbook and a Biomedical Engineering Encyclopedia are being developed; Evidence Based Health Technology; and Medical Equipment Evaluation. IUPESM is establishing collaboration with other members of the ICSU family on these and related projects.

IUPESM is committed to improving public understanding of the applications of science and engineering in health care.

## STRUCTURE

Although the IUPESM currently serves as an umbrella for only two organisations, the IFMBE and the IOMP, it is intended to cover a wide range of technical disciplines in the life sciences, health and healthcare. The provision for the admission of other appropriately qualified international societies is laid down in its constitution. The administrative structure of the Union is shown in Figure 2.The governing bodies of the IUPESM are the General Assembly (GA) and the IUPESM Council [3].

**Figure 1:**
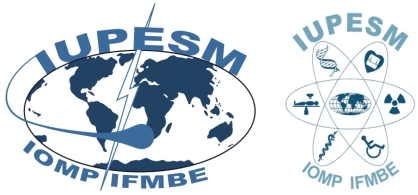
The two IUPESM Logos. The logo on the left is the traditional one which was replaced in 2003 by the new logo on the right. Due to poor visibility of the new logo in electronic publications at small sizes or low resolutions, the old one was reactivated in 2006 as a second choice to be used where appropriate.

**Figure 2:**
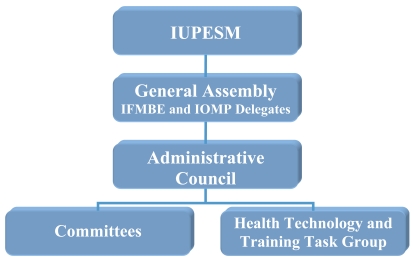
The structure of IUPESM.

The General Assembly, to which the IUPESM Council is responsible, consists of the representatives of the constituent organisations and is the highest authority of the IUPESM. It meets physically every three years during the IUPESM world congresses. The GA determines the Union’s general policy. Its main functions are, among others, to provide guidance for the administration of the IUPESM; to review, accept or reject recommendations of the Council; to elect Officers and Ordinary Members of the IUPESM Council; to amend the statutes and bylaws of the IUPESM; to ratify the creation or dissolution of standing committees, special committees, commissions and other appropriate bodies recommended by the IUPESM Council; and to approve applications for membership.

The activities of the IUPESM are being administered by a Council which consists of the Officers (President, Vice-President, Past President, Secretary General and Treasurer) and Ordinary Members elected by the GA. The President is the retiring President of one of the constituent organisations, and the Vice-President is the retiring President of another of the constituent organisations, with these offices to be alternated between the organisations to give equal representation.

The Council is empowered to act on behalf of the IUPESM but is responsible to the GA for its actions. The Council conducts the business of the IUPESM between sessions of the General Assembly. The Union maintains a number of committees, both standing and ad hoc, to deal with specific and well-defined tasks and fields of particular interest to the Union. Administratively, the Committees are sub-groups of the Council.

Currently there are seven Committees:

- Congress Coordinating Committee, standing
- Nominating Committee, standing
- Awards Committee, standing
- ICSU Liaison Committee, ad hoc
- Education and Training Committee, ad hoc
- Regional Development Committee, ad hoc
- Public and International Relations Committee, ad hoc.

Commissions and Action or Task Groups may be established upon specific needs inside as well as outside the Committee structure, such as the Health Technology and Training Task Group.

**Figure 3:**
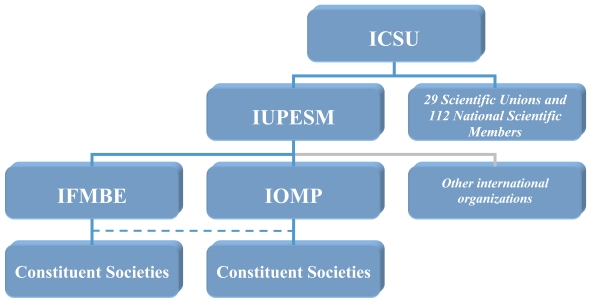
The affiliations of IUPESM.

The affiliations of the Union are shown in Figure 3.

## MEMBERSHIP

Through its adhering national and international societies with members in over 100 countries (see Table 1)the Union comprises a global network of more than 140,000 medical physicists and biomedical engineers dedicated to improving healthcare and people’s well-being worldwide, with some special focus on supporting developing countries in achieving these goals, too. Members of IUPESM have specialised postgraduate training, many with higher degrees. Their activities include research and development that exploits physical and engineering sciences for the maximum benefit of patients and people with disabilities. Scientific support is provided for clinical colleagues in a wide variety of diagnostic and therapeutic procedures and, in some cases, services are provided directly to patients and the disabled.

**Table 1:** International IUPESM member organisations and countries with national member societies.

**IUPESM Membership:** IFMBE and IOMP together are representing national BME and MP societies from 80 countries and 9 international organizations with a total of more than 140,000 individual members in more than 100 countries.
**Countries with national member societies (IOMP and/or IFMBE):** Algeria, Argentina, Australia, Austria, Bangladesh, Belgium, Brazil, Bulgaria, Cameroon, Canada, Chile, China, China Taipei, Colombia, Cuba, Cyprus, Czech Republic, Denmark, Ecuador, Egypt, Emirates, Estonia, Finland, France, Georgia, Germany, Ghana, Greece, Hong Kong, Hungary, Iceland, India, Indonesia, Iran, Ireland, Israel, Italy, Japan, Jordan, Korea, Latvia, Lithuania, Malaysia, Mexico, Moldova, Morocco, Nepal, Netherlands, New Zealand, Nigeria, Norway, Pakistan, Panama, Philippines, Poland, Portugal, Romania, Russia, Serbia, Singapore, Slovakia, Slovenia, South Africa, Spain, Sri Lanka, Sudan, Sweden, Switzerland, Tanzania, Thailand, Trinidad & Tobago, Turkey, Uganda, Ukraine, United Kingdom, United States of America, Venezuela, Zambia, Zimbabwe.
**International member organizations:** Asian-Oceania Federation of Organizations for Medical Physics (AFOMP), Commission for the Advancement of Healthcare Technology Management in Asia (CATHMA), European Alliance for Medical and Biological Engineering (EAMBES), European Federation of Organizations for Medical Physics (EFOMP), European Society for Engineering in Medicine (ESEM), IEEE Engineering in Medicine and Biology Society (IEEE-EMBS), International Council on Medical and Care Compunetics (ICMCC), Latin American Medical Physics Association (ALFIM), Southeast Asian Federation of Organizations for Medical Physics (SEAFOMP).

## AWARDS

Since 1988 the Union presents the IUPESM Awards of Merit every third year. Originally, the award was given in medical physics only, but since 1997 it has been extended to two triennial awards which recognise a medical physicist and a biomedical engineer who have established distinguished careers in medical physics and biomedical engineering, respectively.

## WORLD CONGRESS

IUPESM has held triennial *World Congresses on Medical Physics and Biomedical Engineering* for some 20 years. This is the scientific meeting of the IUPESM and incorporates the international conferences of its affiliate organisations (IFMBE and IOMP), i.e. the International Congress on Medical Physics (ICMP) and the International Congress on Medical and Biological Engineering (ICMBE). The Congress is organised by the national member societies of the country in which the Congress takes place. The Congresses provide a unique opportunity for medical physicists and biomedical engineers to meet and present the latest developments in their respective fields, in an environment that fosters cross-fertilisation of ideas and innovations in these two disciplines.

## ICSU

The most important milestone in the recent history of the IUPESM was its acceptance into the International Council of Science (ICSU) in 1999. ICSU is a non-governmental organisation, founded in 1932, whose mission is to ’strengthen international science for the benefit of society‘. The ICSU membership is made up of 112 national academies of science or research councils and 29 international scientific unions [4].

IUPESM admittance into ICSU, the most prestigious scientific umbrella organisation, represents the true maturing of the biomedical engineering and medical physics professions and their acceptance as equals in the global scientific establishment.

The primary benefits of Full Membership of IUPESM in ICSU are symbiotic for both organisations. ICSU brings to the table its substantial international stature, greater resources, as well as programs and committee structures linking closely to those of the Union. IUPESM offers vast international experience that complements ICSU to create an authoritative international advocate for the application of science, including engineering, for the benefit of the sick and disabled worldwide. In addition IUPESM contributes its key programs outlined earlier in this document, all with special reference to developing countries and to be linked with ICSU's committees on science and developing countries as well as its programs on capacity building in science and its International Network for the Availability of Scientific Publications.

There are currently many organisations that work towards improving health and well-being. However, there is a lack of coordination at the global level between the basic sciences and the medical community. Moreover, the wealth of scientific knowledge is often not fully utilised to address the human condition. IUPESM believes that science should be more effectively employed worldwide to improve health and well-being. In 2002, both IUPESM and IFMBE appointed Prof Dov Jaron (Drexel University, Philadelphia) to assume a leading role in establishing a major trans-ICSU Unions initiative on ’Science for Health and Well-Being’ (SHWB). Dov Jaron proposed the formation of this initiative at the ICSU General Assembly in Rio, during a meeting of ICSU’s bio-related Unions, and participated in extensive discussions that followed its creation. These discussions brought about a major addition to the 2006-2011 Strategic Plan for ICSU together with a new emphasis on health “to ensure that health considerations are duly taken into account in the planning and execution of future activities (of ICSU).”

The mission and scope of the SHWB initiative were defined as follows: the Science for Health and Well-Being Initiative (SHWB) seeks to improve and advance human health and well-being. It will marshal expertise from the natural, social, behavioural and engineering sciences into a coordinated program designed to attain new insights into research and policies that affect the health and well-being of people, mindful always of their intimate links to the health of the fauna, flora, and environmental systems among which humankind abides. SHWB will organise programs and projects that transcend scientific boundaries and are most effectively pursued by multidisciplinary teams, including teaching and research units to explore real life problems of various communities. SHWB will identify unmet needs and work within a distinctive conceptual niche that complements research conducted in other international initiatives. SHWB will build upon existing synergies among international scientific organisations affiliated with the International Council for Science (ICSU), seek to foster new synergies among them, and welcome links with other international programs.

Seventeen ICSU Unions and a number of ICSU Interdisciplinary bodies have joined the initiative and will participate in various programs and policy matters related to health and well-being. IUPESM, as a Union most closely related to the new strategic focus of ICSU, will retain a leading role in ICSU’s programs on human health.

## FUTURE TASKS AND GOALS: APPROPRIATE HEALTH TECHNOLOGIES FOR DEVELOPING COUNTRIES

Modern healthcare relies heavily on a whole range of health technologies [5]. Health technologies should be efficient, safe, cost-effective and available to all people without causing an excessive financial burden to the healthcare systems, in order for the technologies to be achievable and sustainable. An important prerequisite to attaining these goals is the implementation of health technology assessment, planning and management as an accountable, systematic approach to ensuring that the technologies meet the demands of high quality patient care.

Health technology assessment (HTA) has, in times of rising costs of healthcare systems and limited budgets in most countries, become an important tool and often a political issue, too. The rapid growth of medical knowledge as well as diagnostic and therapeutic techniques and technologies requires a conscientious employment of available resources. Finding appropriate solutions is a major challenge for HTA since a wide range of aspects has to be included into the decisions, such as security, efficacy, cost in comparison to the benefit, as well as social, legal and ethical implications. Decisions in the health sector and in health policy are to be made on the basis of scientific findings, meaning that they have to be evidence-based. HTA helps to prevent the uncontrolled dissemination of unsuitable technologies in the health systems, as well as to minimise the financial burden involved and to increase the quality of health care. With an early comprehensive evaluation, HTA also contributes to a fast integration of innovative procedures in the health systems, as well as to a removal of unnecessary, and therefore cost-intensive, methods.

In spite of all efforts to make the whole range of health technologies available to every country, an increasing number of nations still cannot shoulder the financial burden of acquiring and maintaining all technologies that would be desirable and beneficial for the healthcare of their people. Therefore it is necessary to establish priorities based on available resources and the burden of disease, a rather complex task for which the World Health Organization (WHO) together with the IFMBE has already developed methodologies and tools such as the WHO Integrated Health Technology Package (IHTP) [6].

One of the prerequisites for proper use of health technologies is the existence of an appropriate, reliable infrastructure. In order to set up and/or maintain such an infrastructure, centres for health technologies should be established as part of the health ministries or at least strongly linked to them. These centres should implement the national strategies and plans for the health technologies, as well as oversee and guide the national healthcare systems and, where appropriate, regional healthcare centers with regard to the health technologies. These centres should also collaborate and build partnerships with healthcare providers, industry, patients’ associations and professional, scientific and technical organisations.

Another important step in improving the quality of healthcare through health technologies is to build up the necessary health workforce, i.e. medical physicists, clinical engineers and technicians, which will be able to manage, maintain and operate the technologies and educate the users, i.e. physicians and nurses, in the safe and competent use of equipment and devices. The industrialised countries should be called upon to offer educational support to help those countries who cannot afford to provide education and training for a sufficient number of clinical engineers and technicians.

The IUPESM in cooperation with the WHO has set up a joint IFMBE/IOMP Health Technology and Training Task Group (HTTTG), directly responsible to the IUPESM Council. This task group will assist countries in defining their health technology needs, and identifying and rectifying health system constraints for adequate management and utilisation of health technology, particularly through training, capacity-building and the development and application of appropriate technology. Specific tasks for the Group are to help identify needs in health technologies and training for each cooperating country, to make recommendations for actions to satisfy these needs as far as appropriate and possible, to support the countries in the necessary actions, and to set up a brokerage to match health technology donations with appropriate recipients.
